# Critical evaluation of a crystal structure of nitrogenase with bound N_2_ ligands

**DOI:** 10.1007/s00775-021-01858-8

**Published:** 2021-03-13

**Authors:** Justin Bergmann, Esko Oksanen, Ulf Ryde

**Affiliations:** 1 Department of Theoretical Chemistry, Lund University, Chemical Centre, P. O. Box 124, 221 00 Lund, Sweden; 2 European Spallation Source ESS ERIC, Lund, Sweden

**Keywords:** Nitrogenase, Quantum refinement, N_2_ binding, Reaction intermediates

## Abstract

Recently, a 1.83 Å crystallographic structure of nitrogenase was suggested to show N_2_-derived ligands at three sites in the catalytic FeMo cluster, replacing the three $\mu_{2}$ bridging sulfide ligands (two in one subunit and the third in the other subunit) (Kang et al. in Science 368: 1381–1385, 2020). Naturally, such a structure is sensational, having strong bearings on the reaction mechanism of the enzyme. Therefore, it is highly important to ensure that the interpretation of the structure is correct. Here, we use standard crystallographic refinement and quantum refinement to evaluate the structure. We show that the original crystallographic raw data are strongly anisotropic, with a much lower resolution in certain directions than others. This, together with the questionable use of anisotropic B factors, give atoms an elongated shape, which may look like diatomic atoms. In terms of standard electron-density maps and real-space *Z* scores, a resting-state structure with no dissociated sulfide ligands fits the raw data better than the interpretation suggested by the crystallographers. The anomalous electron density at 7100 eV is weaker for the putative N_2_ ligands, but not lower than for several of the ${\mu }_{3}$ bridging sulfide ions and not lower than what can be expected from a statistical analysis of the densities. Therefore, we find no convincing evidence for any N_2_ binding to the FeMo cluster. Instead, a standard resting state without any dissociated ligands seems to be the most likely interpretation of the structure. Likewise, we find no support that the homocitrate ligand should show monodentate binding.

**Supplementary Information:**

The online version contains supplementary material available at 10.1007/s00775-021-01858-8.

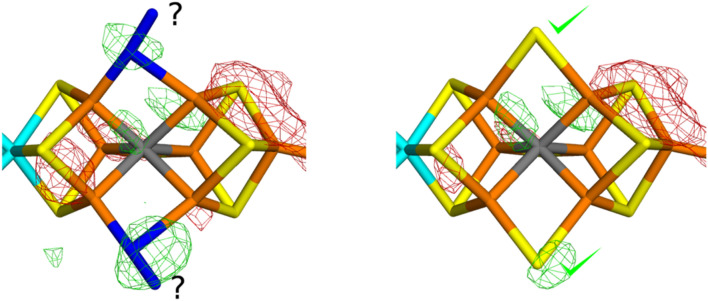

## Introduction

Nitrogenase is the only enzyme that can catalyse the cleavage of the strong triple bond in N_2_, thereby making nitrogen available for plants [1]. Nitrogenase reduces N_2_ to ammonia, through the reaction:

$${\text{N}}_{2} + 8 e^{ - } + 8 {\text{H}}^{ + } + 16 {\text{ATP}} \to 2 {\text{NH}}_{3}^{ + } + {\text{H}}_{2} + 16 {\text{ADP}} + 16 {\text{P}}_{i}$$

The mechanism is normally discussed in terms of the eight-state Thorneley–Lowe cycle, involving states *E*_0_ to *E*_7_, differing in the number of added electrons and protons [2, 3]. *E*_0_ is the resting state and it is currently believed that N_2_ binds to *E*_4_ with the concomitant release of H_2_ through reductive elimination of two hydride ions [1].

**Fig. 1:**
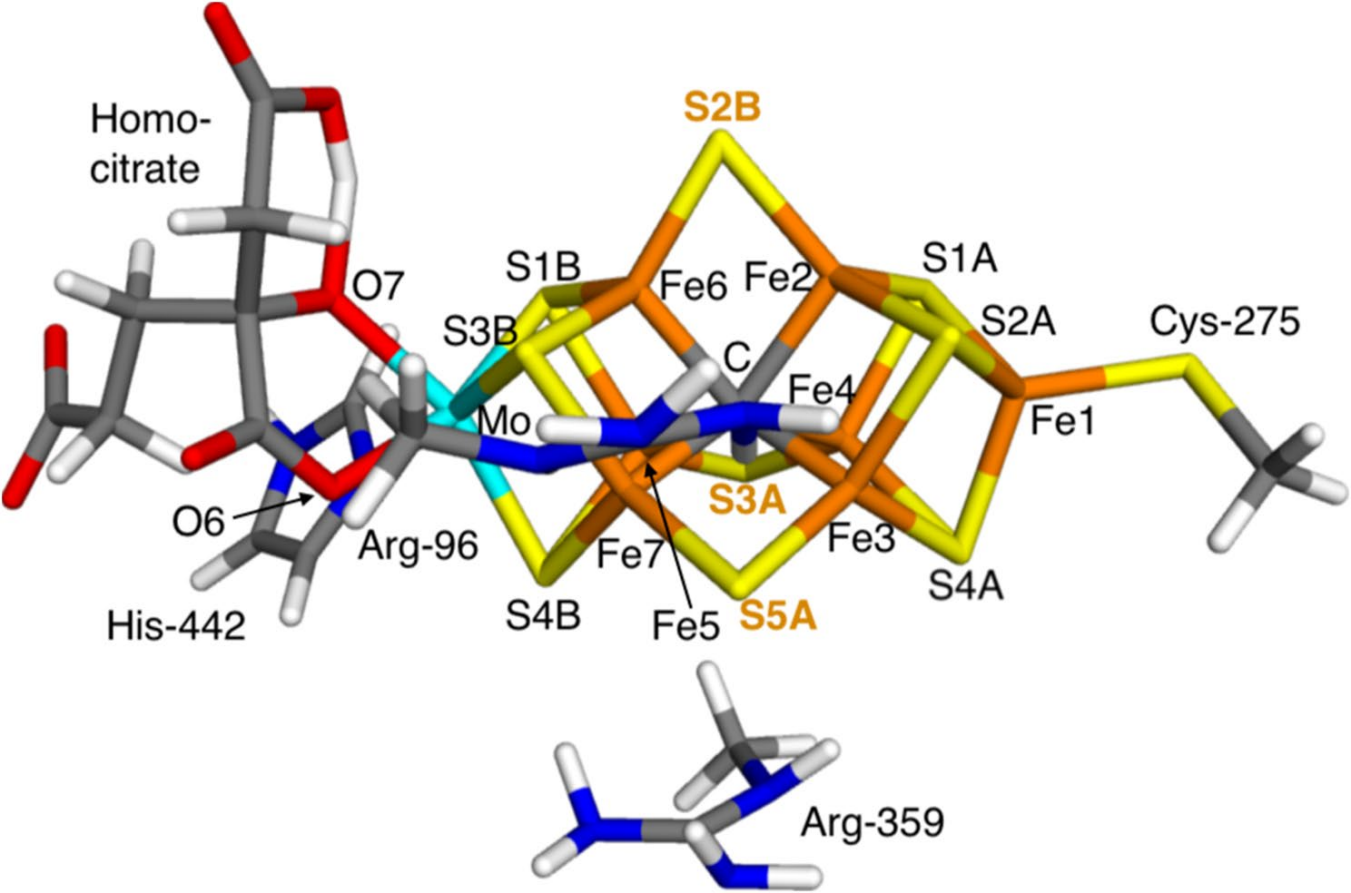
The FeMo cluster in nitrogenase illustrating the atom names and also the QM system in the quantum-refinement calculations. The three ${\mu }_{2}$ bridging sulfide ions are emphasised with bold orange text

Several crystal structures have shown that the active site is a complicated MoFe_7_S_9_C(homocitrate) cluster [4, 5], the FeMo cluster, shown in Fig. 1. It is essentially composed of two merged Fe_4_S_4_ cubane clusters (one with a Mo substitution), connected by three $\mu_{2}$ bridging sulfide ions and a central carbide ion. The homocitrate ligand binds bidentately to Mo and the cluster is connected to the protein by a histidine ligand to Mo and a single cysteine ligand binding to the terminal Fe ion.

A problem with the mechanistic understanding of nitrogenases has been that it is not clear where the N_2_ substrate binds – there is no open coordination site or any labile ligands. However, crystal structures of CO-inhibited nitrogenases have shown that CO replaces one of the $\mu_{2}$ bridging sulfide ions (S2B; atom labels are shown in Fig. 1), bridging Fe2 and Fe6 [6]. A similar replacement was also observed for a crystal structure of a turnover state, which was originally interpreted as showing a N_2_-derived reaction intermediate [7], but later studies have shown that it probably contains an OH^−^ ion instead [8, 9]. This has inspired computational investigations of reaction mechanisms involving the exchange of S2B with the N_2_ substrate [10, 11].

Recently, a 1.83 Å crystal structure of nitrogenase was presented, obtained under physiological N_2_ turnover conditions [12]. The authors suggested that in one of FeMo clusters in the dimeric enzyme, the S2B ligand is replaced by N_2_, whereas in the other FeMo cluster, the other two $\mu_{2}$ sulfide ligands (S3A and S5A) are replaced by N_2_ (or possibly N_2_H_2_ or N_2_H_4_). The suggestions were supported by anomalous density maps measured at 7100 eV, showing reduced densities for the dissociated sulfide ligands, as well as elongated omit maps, indicating diatomic molecules, rather than the spherical sulfide ions. Based on these findings, the authors suggested that all these three sites are employed in the reaction mechanism (possibly by the rotation of the whole cluster), taking advantage of the differing surroundings that may provide protonation at different positions of the substrate or intermediates.

Of course, such suggestions are sensational and would have a strong impact on the understanding of nitrogenase. Therefore, it is important to ensure that the interpretation of the crystal structure is correct and better than alternative interpretations. In this study, we provide a thorough evaluation of the crystal structure with both standard crystallography means and by quantum refinement [13]. We show that the crystallographic raw data are quite poor, with a strong anisotropy. The arguments for replacement of the three $\mu_{2}$ sulfide ligands are weak and a model of the *E*_0_ resting state with all sulfide ligands bound to the cluster fits the data at least as good as the structure presented in the original publication.

## Methods

### Crystal structure

This study is based on the 6UG0 crystal structure of Mo nitrogenase at 1.83 Å resolution [12]. Coordinates, occupancies, B factors and structure factors were obtained from the Protein Data Bank, together with the space group, unit-cell parameters, resolution limits, $R$ factors and the test set used for the evaluation of the *R*_free_ factor. For the evaluation of the deposited structure, the electron-density map coefficients were also downloaded from the Protein Data Bank. The anomalous electron density map was downloaded from Zenodo [14].

### Quantum refinement

In standard crystallographic refinement, the current model (coordinates, B factors, occupancies, etc.) is optimised by minimising the difference between structure factors observed experimentally or calculated from the model [15]. Owing to the limited resolution of protein crystal structures, it is normally necessary to introduce restraints in the crystallographic refinement to ensure that the structure makes chemical sense. These restraints are usually derived from high-resolution structures [16] and in the language of computational chemistry, they represent a molecular-mechanics (MM) force field. Therefore, the refinement optimises an energy function of the form

1 $$E_{{{\text{cryst}}}} = w_{{\text{A}}} E_{{{\text{Xray}}}} + E_{{{\text{MM}}}}$$

Here, *E*_Xray_ is the crystallographic goodness-of-fit criterion, typically a maximum-likelihood function [17, 18], *E*_MM_ is the empirical restraints and *w*_A_ is a weight factor determining the relative importance of the two terms.

The empirical restraints are most accurate for protein residues and nucleic acids, for which there are much accurate experimental data. However, for cofactors, substrates and inhibitors, much less information is available, making the restraints less certain [19]. Even worse, for metal sites, it is hard to set up an empirical potential [20] and it depends strongly on all the ligands, as well as the charge and spin state of the metal. Therefore, these parts of crystal structures have a lower accuracy than the amino-acid parts.

To overcome these problems, the empirical restraints can be replaced by quantum–mechanical (QM) calculations. This can be done for a small, but interesting part of the structure (e.g. the active site) in the same way as in standard QM/MM methods [21, 22]. This part is called system 1 in the following. This leads to the quantum-refinement energy function [13].

2 $$E_{{{\text{cqx}}}} = w_{{{\text{MM}}}} \left( {w_{{\text{A}}} E_{{{\text{Xray}}}} + E_{{{\text{MM}}}} - E_{{{\text{MM1}}}} } \right) + E_{{{\text{QM1}}}}$$

Here, *E*_QM1_ is the QM energy of system 1. To avoid double-counting of energy terms, we need to subtract the corresponding MM energy of system 1, *E*_MM1_. *w*_MM_ is another weight factor that is necessary because the empirical restraints are normally in statistical units, whereas the QM energy is in energy units.

Such an energy function is implemented in the ComQum-X software [13], which is an interface between the QM software Turbomole [23] and the software crystallography and NMR system (CNS) [24, 25]. We employed the default *w*_A_ factor, selected by CNS, 1.5368. Likewise, we used *w*_MM_ = 1/3 as in all our previous applications [13].

### QM calculations

The QM calculations were performed at the TPSS/def2-SV(P) level of theory [26, 27]. The calculations were sped up by expanding the Coulomb interactions in an auxiliary basis set, the resolution-of-identity (RI) approximation [28, 29]. Empirical dispersion corrections were included with the DFT-D4 approach [30, 31]. We studied the FeMo clusters in both the A and C subunit of the protein. In both cases, the QM systems were FeMo cluster, homocitrate, the imidazole ring from His-442, the side chain of Cys-275 and the side chains of Arg-96 and Arg-359 (modelled as CH_3_NHC(NH_2_)_2_^+^). The two Arg residues where included to compensate the negative charge of the cluster. The QM system is shown in Fig. 1.

In the $E_{0}$ resting state of Mo nitrogenase, the FeMo cluster is in the Mo(III)Fe(II)_3_Fe(III)_4_ oxidation state [32, 33]. This gives a net charge of –3 for the QM system in Fig. 1. It is normally assumed that this charge is conserved throughout the Thorneley–Lowe reaction cycle, because each added electron is accompanied by a proton. However, when a sulfide ion dissociates, it takes two negative charges with it (S^2−^), so that the net charge of the cluster increases by two for each dissociated sulfide. Therefore, we have assumed that structures with one sulfide ion displaced by N_2_ has a net charge of − 1, whereas models with two N_2_ molecules have a net charge of + 1. On the other hand, we assumed that systems with N_2_ and N_2_H_2_ have the same net charge (because they represent two different $E_{n}$ states, viz. after the addition of two electrons and protons). In three cases, we tested also a net charge of − 3 for the N_2_-bound systems, i.e. assuming that the net charge of the cluster is conserved also after the dissociation of the sulfide ion. That would correspond to a formal reduction of two Fe ions. All structures were studied in the quartet state, which is the observed spin state for $E_{0}$ [1].

The electronic structure in all QM calculations was obtained with the broken-symmetry approach [34]: each of the seven Fe ions were modelled in the high-spin state, with either a surplus of $\alpha$ (four Fe ions) or $\beta$ (three Fe ions) spin. We employed the broken-symmetry BS7-235 state with $\beta$ spin on Fe2, Fe3 and Fe5 for all calculations (the numbering of the Fe ions is shown in Fig. 1). This is the best broken-symmetry state for the resting state of Mo nitrogenase and also for several other $E_{n}$ states [34–36]. This state was obtained using the fragment approach by Szilagyi and Winslow [37] or by swapping the coordinates of the Fe ions [38].

## Result and discussion

We have performed a critical evaluation of the recent crystal structure of nitrogenase (6UG0 at 1.83 Å resolution) [12], suggested to show that in chain A, one of the $\mu_{2}$ bridging belt sulfide ions of the FeMo cluster (S2B) is replaced by a N_2_ ligand (possibly protonated), whereas in chain C, instead the other two $\mu_{2}$ bridging sulfide ions (S3A and S5A) are replaced by N_2_. We employ standard crystallographic metrics (electron-density maps and RSZD scores), as well as quantum refinement to evaluate the structure and study whether there are any convincing arguments that the sulfide ions really are replaced by N_2_. In the following we will call the three sites 2B, 3A and 5A, corresponding to the binding sites of S2B, S3A and S5A, respectively (Fig. 1), even when N_2_ is binding in that site.

### The original crystal structure

**Fig. 2:**
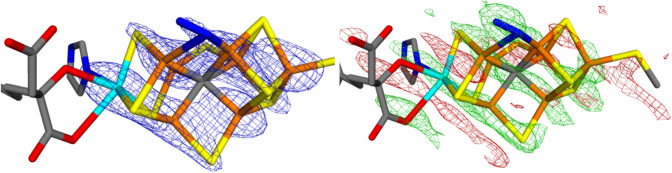
The deposited 2*mF*_o_–*DF*_c_ (7 σ; left) and *mF*_o_–*DF*_c_ maps (right; + 3.0 σ in green and − 3.0 σ in red) around the active-side MoFe cluster in the A chain of Mo nitrogenase

We start by describing the deposited structure and the corresponding electron-density maps (downloaded from the PDB server, https://www.rcsb.org/structure/6UG0). The 2*mF*_o_–*DF*_c_ electron-density map of the FeMo cluster in chain A is shown in Fig. 2a. The Fe ion peaks start to be visible in the 2*mF*_o_–*DF*_c_ map at 15.4 $\sigma$, but the weakest one (Fe) appears at 10.9 $\sigma$. The S ion peaks start appearing at 8.4 $\sigma$ while the putative N_2_ ligand is a $\sim$ 6.8 $\sigma$ peak. On the other hand, S1A in the same cluster is only a 5.7 $\sigma$ peak and the Cys sulfur a 5.3 $\sigma$ peak, so the 2B site does not have the lowest electron density among the S ions in the cluster. In fact, the 2*mF*_o_–*DF*_c_ electron-density map shows a conspicuous layered structure and sites outside these layers seem to have a lower electron density.

To obtain more robust measures of the density peak volumes, we have integrated the 2*mF*_o_–*DF*_c_ electron density around each S atom in the crystal structure within a sphere with a radius of 1.05 Å (the covalent radius of S). The results are shown in Table 1. It can directly be seen that the integrated density at the 2B site is not particularly low, neither when compared to all other S atoms in the crystal structure (+ 0.6 $\sigma$, i.e. larger than the average) nor when compared to all the other S atoms in the cluster (close to the average value).

**Table 1:** Integrated electron densities for all S atoms in the two FeMo clusters in nitrogenase, using the 2*mF*_o_–*DF*_c_ map of the original crystal structure (but with the putative N_2_ molecules replaced by an S atom at a position taken from the quantum-refined structures when integrating the electron densities)

Atom	Chain A			Chain C		
	*ρ*	*ρ*_all_	*ρ*_FeMo_	*ρ*	*ρ*_all_	*ρ*_FeMo_
Cys	19.2	0.14	− 1.09	21.7	0.62	− 1.69
S2B	21.6	0.60	0.02	23.6	1.01	− 0.94
S3A	21.7	0.64	0.10	25.4	1.36	− 0.26
S5A	20.4	0.37	− 0.54	25.2	1.32	− 0.33
S1A	17.8	− 0.13	− 1.73	29.0	2.06	1.12
S2A	20.9	0.48	− 0.27	27.1	1.69	0.39
S4A	25.3	1.35	1.79	27.2	1.71	0.44
S1B	22.3	0.74	0.35	30.5	2.37	1.73
S3B	22.4	0.77	0.40	26.6	1.60	0.22
S4B	23.6	1.00	0.97	24.3	1.14	− 0.68
Av		18.5	21.5		18.5	26.1
SD		5.1	2.1		5.1	2.6

*ρ* is the raw integrated electron density within a sphere of 1.05 Å radius, in units of $e$. In *ρ*_all_ and *ρ*_FeMo_, this density is presented in $\sigma$ units compared to the average and standard deviation over all S atoms in the crystal structure or the S atoms in the same cluster, respectively (the corresponding average and standard deviations are given in the last two lines of the table)

The layered structure becomes even clearer when considering the *mF*_o_–*DF*_c_ electron-density difference maps (Fig. 2b), showing alternating layers of positive and negative densities. In particular, there is strong positive densities at almost all atoms in the FeMo cluster and strong negative density between the atoms. The positive density is highest close to the central carbide ion (6.7 $\sigma$), but it is high also close to the N_2_ ligand (6.6 $\sigma$), indicating that the N_2_ model contains too few electrons (N_2_ contains 14 electrons, whereas S^2–^ contains 18 electrons). The largest positive density at any other S atom of the FeMo cluster appears at $\sim$ 5.6 $\sigma$ (but there is a layer of positive density between Fe7 and S5A at 6.1 $\sigma$.

**Fig. 3:**
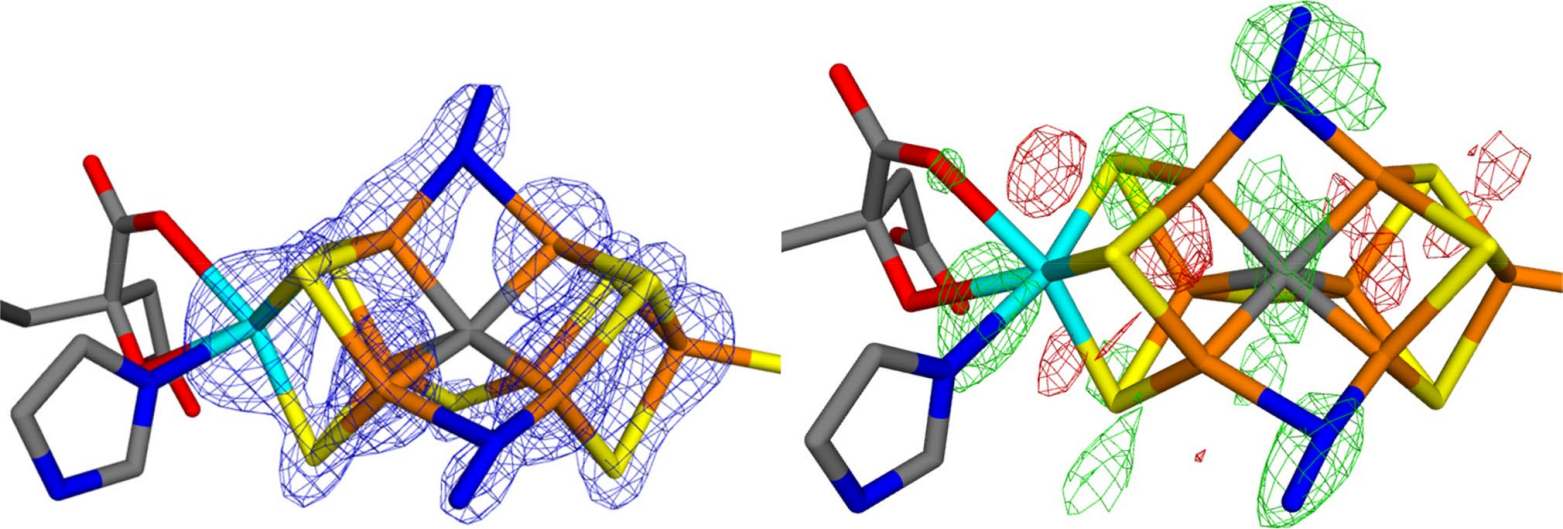
The deposited 2*mF*_o_–*DF*_c_ (7.6 σ; left) and *mF*_o_–*DF*_c_ maps (right; + 5.0 σ in green and − 5.0 σ in red) around the active-side MoFe cluster in the C chain of Mo nitrogenase

In chain C, the crystal structure suggests that S2B is present, but both S3A and S5A are replaced by N_2_ (again possibly protonated). Figure 3a shows the 2*mF*_o_–*DF*_c_ electron density of the FeMo cluster in chain C. The Fe ion peaks start to appear at 15.5 $\sigma$ and all are visible at 13.6 $\sigma$. The sulfide ions start to appear at 9.7 $\sigma$ (S2A). There is electron density at the N_2_ ligand in the 5A site already at 9.3 $\sigma$, when several sulfide ions are still not seen, including S2B. Density is seen at the other N_2_ ligand at 8.6 $\sigma$ and at the same level also S2B starts to appear. S4B does not appear until 7.5 $\sigma$. The integrated densities show that the 3A and 5A sites actually have high densities compared to all S atoms (1.3–1.4 $\sigma$), but slightly lower than for the $\mu_{3}$ bridging sulfide ions (–0.3 $\sigma$ compared to all S atoms in the cluster), but the deviation is very small (the S2B ion actually has a much smaller integrated density). Thus, there is no indication from the electron density that S3A and S5A have been replaced by other ligands.

The *mF*_o_–*DF*_c_ electron-density difference maps of the FeMo cluster in chain C are shown in Fig. 3b. They also show a layered structure and large difference densities in the FeMo cluster. The largest positive density is found between Mo and the OH group of the homocitrate ligand (8.9 $\sigma$), but there are also large positive densities both at the N_2_ ligand in the 3A site and between Fe7 and S3B ($\sim$ 8.0 $\sigma$). A positive density appears at the other N_2_ ligand at 7.7 $\sigma$. None of the other S ligands have any positive density until $\sim$ 3 $\sigma$.

The prominent layer-like features of the electron density can be linked to a strong anisotropy of the data. In fact, the data extends to the reported 1.83 Å resolution along the c*-axis, but along the b*-axis the resolution is only $\sim$ 2.6 Å according to the PDBPeep server [39]. Since $I{/}\sigma \left( I \right) > 5$ in the best direction at the 1.83 Å resolution cutoff, it cannot be excluded that the layers are anisotropic Fourier ripples around the electron rich FeMo cluster. There are also missing wedges of data around the a* and c* axes, which further degrades the quality of the data set.

Interestingly, the original authors selected to refine the structure with anisotropic B factors, although the resolution is only 1.83 Å. This is strongly questionable, especially given the anisotropy of the data. Even with isotropic data to 1.83 Å, the use of anisotropic B factors would hardly be standard, but the marked data anisotropy causes an unphysical systematic effect in the B factors. Figure S2 in the supplementary material indeed shows that all B factors are strongly anisotropic, in contrast to normal high-resolution structures, in which most atoms have almost spherical B factors. The anisotropy follows the layers in the difference density, implying that the two phenomena are connected. Naturally, this will strongly affect the interpretation of the structure, in particular whether the replaced sulfide ligands are interpreted as a single atom or two N atoms. In that regard, it is important to note that the two N atoms of the N_2_ ligands always lie along the axis of maximum anisotropy. This indicates that the interpretation of the ligands as diatomic ligand may actually be an artefact of the anisotropy of the data.

To avoid this bias, we refined the structure with only isotropic B factors in the following sections, using default settings with the Phenix software.

### Quantum refinement

Next, we used the method of quantum refinement to test different models of the crystallographic data, viz. testing either S^2–^, N_2_ or N_2_H_2_ in the 2B (chain A) or 3A and 5A (chain C) binding sites. Quantum refinement is standard crystallographic refinement in which the empirical restraints are replaced by accurate QM calculations for a small (but interesting) part of the structure. Thereby, we introduce information of the expected structure with different sets of ligands, which may help the interpretation of the structure. We judge the results in terms of the real-space *Z* score (RSZD) for the various parts of the QM system (other atoms are kept at the original crystal structure) and the *mF*_o_–*DF*_c_ electron-density difference maps (obtained using Phenix without any anisotropic B factors).

The RSZD scores for chain A are shown in Table 2. It can be seen that a S^2–^ ligand gives slightly smaller RSZD scores than a N_2_ ligand. In particular, the RSZD score around the 2B site is 2.1 for S^2–^ but 2.7 for N_2_. Moreover, the sum of the RSZD scores of all atoms in the QM system are 21.8 for S^2–^, but 23.0 for N_2_. Changing the net charge of the QM system for the N_2_-bound model to − 3 (i.e. the same as for the S^2–^-bound model instead of − 1, has only a small effect on the RSZD scores, but gives slightly worse results (the sum of the RSZD scores increases to 23.7).

**Table 2:** Results for the quantum refinements of chain A of Mo nitrogenase with different interpretations of site 2B (sulfide or N_2_)

2B	$q$	Arg 96	Cys 275	Arg 359	His 442	HCA 601	FeMo	2B	Sum
S^2–^	− 3	0.2	0.3	0.7	1.0	6.6	10.9	2.1	21.8
N_2_	− 1	0.2	0.4	0.6	1.0	6.9	11.2	2.7	23.0
N_2_	− 3	0.2	0.3	0.6	1.1	6.6	11.4	3.5	23.7
Original		0.1	1.2	0.3	1.1	5.2	21.4	4.5	33.8

The structures are evaluated in terms of the real-space *Z* score based on the difference maps (RSZD). The last column shows the sum of the RSZD scores in the other seven columns. The last line shows the corresponding results in the original crystal structure (obtained from the electron-density maps downloaded from PDB) [12]. $q$ is the net charge of the QM system

**Fig. 4:**
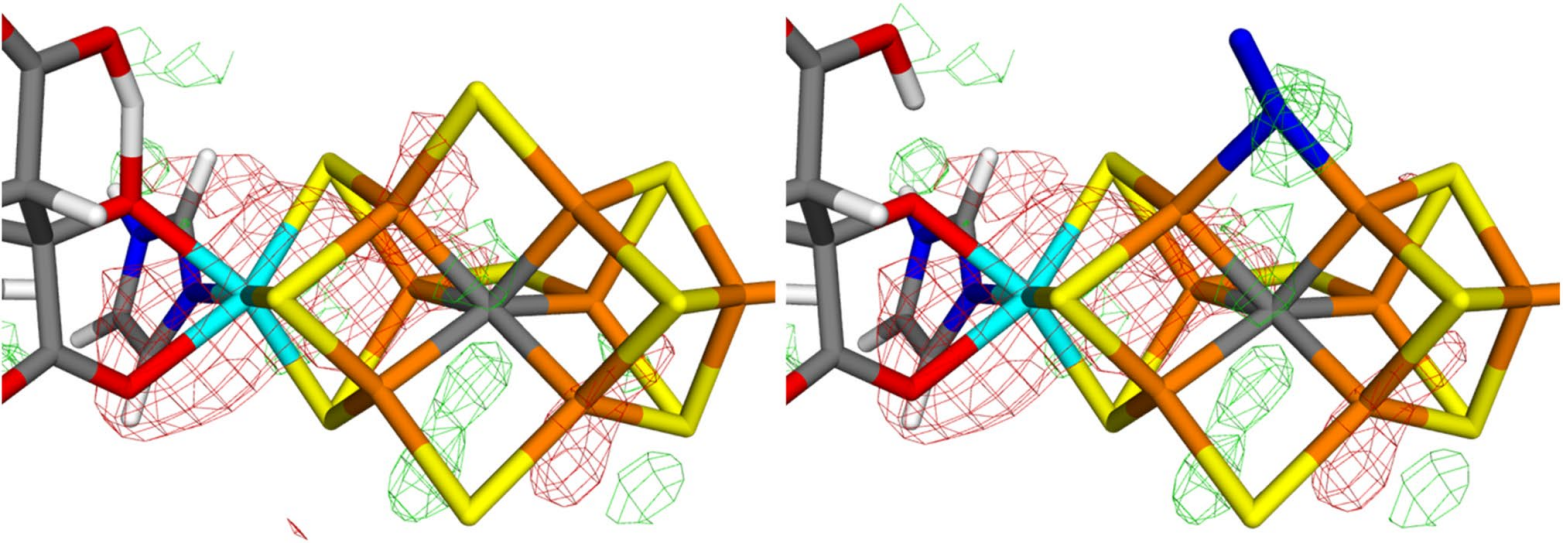
Electron-density difference maps around the MoFe cluster (chain A) of Mo nitrogenase modelled with either S^2−^ (left) or N_2_ (right) in the 2B site. The *mF*_o_–*DF*_c_ difference maps are contoured at + 3 σ (green) and − 3 σ (red)

This interpretation is also confirmed by the *mF*_o_–*DF*_c_ electron-density difference maps for the FeMo cluster in chain A with the 2B site modelled either with S^2−^ or N_2_, shown in Fig. 4. It can be seen that the N_2_ ligand gives rise to a large positive density around the ligand, indicating that it contains too few electrons. On the other hand, the S^2−^ ligand does not show any negative density, although there are some enhanced negative densities around the Fe6 ion and in the direction towards S2B.

For chain C, we performed six different quantum-refinement calculations employing different ligands in the 3A and 5A binding sites, viz. either S^2−^, N_2_ or N_2_H_2_. The results in Table 3 show that the model with S^2–^ in both sites clearly gives the best results. In particular, the sum of the RSZD scores of the QM system is 16.3, whereas the other five models have sums of 18.2–23.3, with the structure using N_2_ in both sites (i.e. the interpretation in the original crystal structure) giving the worst results. Likewise, it can be seen that all models with S^2–^ in site 5A give an appreciably lower RSZD score for site 5A (1.1–1.4), than N_2_ (3.2–3.7), and that N_2_H_2_ gives the worst results (5.7). The results are similar (but slightly worse) in site 3A: all models with S^2–^ give lower RSZD (2.8–3.1) than N_2_ (5.1–6.3) or N_2_H_2_(6.7).

**Table 3:** Results of the quantum refinements for chain C of Mo nitrogenase with different interpretation of the ligands in the 3A and 5A sites (S^2−^, N_2_ or N_2_H_2_)

3A	5A	$q$	Arg 96	Cys 275	Arg 359	His 442	HCA	FeMo	3A	5A	Sum
S^2–^	S^2–^	− 3	0.3	0.1	1.5	0.7	1.6	7.7	3.0	1.4	16.3
S^2–^	N_2_	− 1	0.3	0.1	1.3	0.6	1.7	8.5	2.9	3.3	18.7
S^2–^	N_2_	− 3	0.4	0.1	1.4	0.7	1.6	7.2	3.1	3.7	18.2
S^2–^	NNH_2_	− 1	0.3	0.1	1.3	0.7	1.7	8.9	2.8	5.7	21.5
N_2_	S^2–^	− 1	0.4	0.1	1.6	0.5	1.8	8.6	6.3	1.2	20.5
N_2_	S^2–^	− 3	0.3	0.1	1.4	0.5	1.8	8.6	5.1	1.3	19.1
NNH_2_	S^2–^	− 1	0.3	0.1	1.6	0.5	1.8	8.4	6.7	1.1	20.5
N_2_	N_2_	+ 1	0.5	0.1	1.6	0.7	1.8	9.3	6.1	3.2	23.3
original structure			0.2	2.8	1.1	1.2	6.9	18.4	8.5	8.8	47.9

The structures are evaluated in terms of the RSZD factor. The last column shows the sum of the RSZD scores in the other eight columns. The last line shows the corresponding results in the original crystal structure (obtained from the electron-density maps downloaded from PDB) [12]. $q$ is the net charge of the QM system

**Fig. 5:**
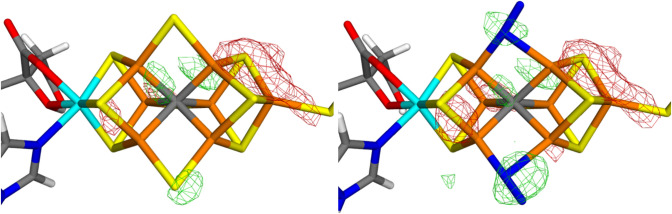
Electron-density difference maps around the active-side MoFe cluster in chain C of Mo nitrogenase modelled with either two molecules of S^−2^ (left) or two molecules of N_2_ (right) in the 3A (up) and 5A sites (down). The *mF*_o_–*DF*_c_ difference maps are contoured at + 3 σ (green) and − 3 σ (red)

This is also supported by the *mF*_o_–*DF*_c_ electron-density difference maps in Fig. 5, showing that the model with two N_2_ ligands give significant positive densities for the two N_2_ ligands, indicating that they contain too few electrons. On the other hand, if we instead use two S^2–^ ligands, no negative difference densities are seen around the ligands in the 3A and 5A sites, which would indicate that they contain too many electrons (there is instead still some positive density around the S3A ion). *mF*_o_–*DF*_c_ difference maps for the other quantum-refined structures are given in the supplementary material.

Thus, the quantum-refinement calculations give no support to the suggestion that some of the $\mu_{2}$ sulfide ligands are replaced by N_2_ in any of the two chains. On the contrary, a normal cluster with all sulfide ligands remaining gives appreciably better results.

### Anomalous densities

**Fig. 6:**
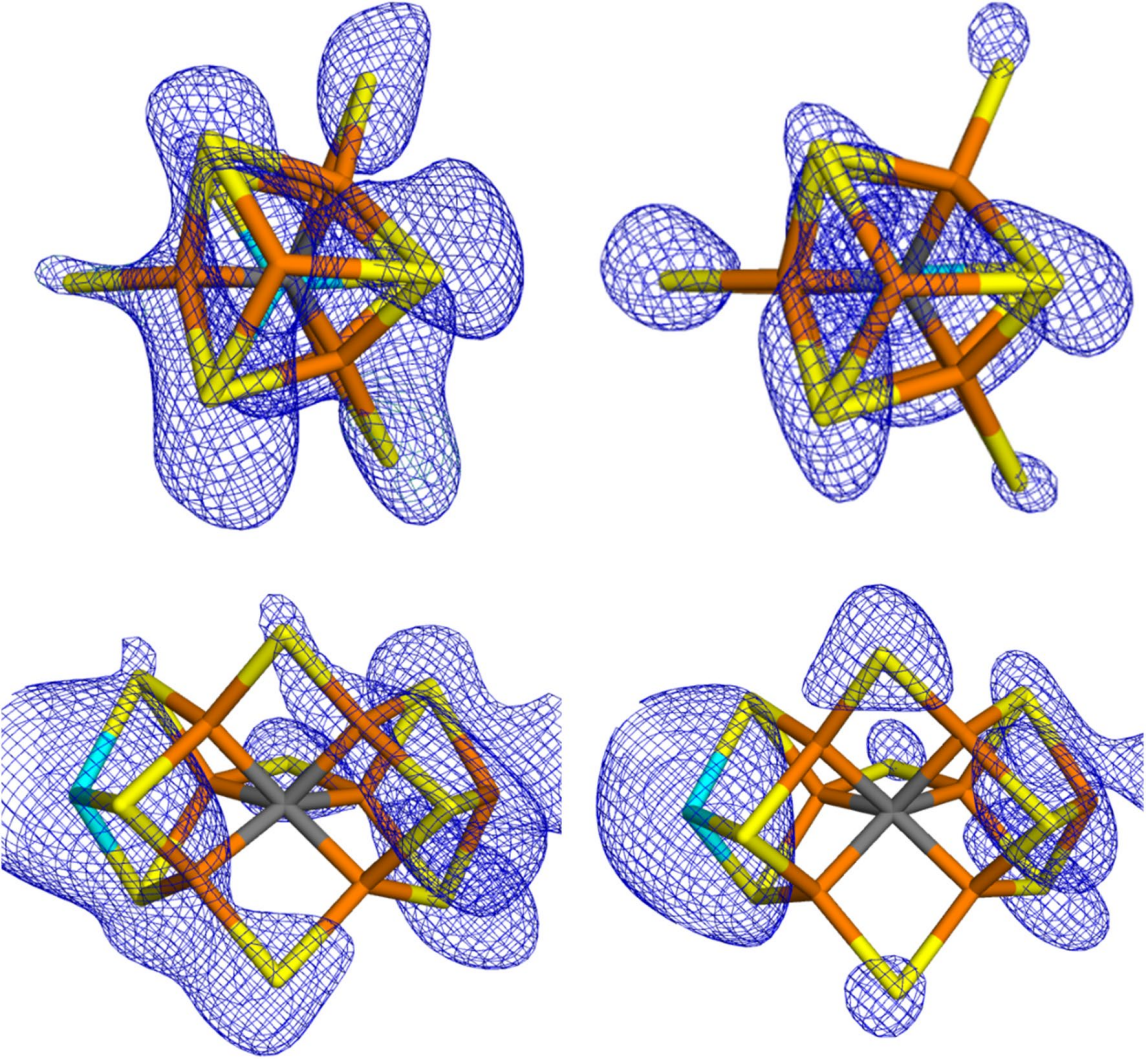
Anomalous-density 2*mF*_o_–*DF*_c_ maps around the active-side MoFe cluster (left: chain A at 3.3 σ and right: chain C at 4.4 σ) of Mo nitrogenase. In the upper figures, S2B points to the left, S3A points up and S5A points down. The two lower figures show a transverse view, like the one in Fig. 1

The strongest argument for the replacement of the $\mu_{2}$ belt sulfide ions came from an analysis of the anomalous densities [12]. Electron-density maps collected at 7100 eV show signals that mainly reflect sulfur and molybdenum atoms. Figure 6 shows the 2*mF*_o_–*DF*_c_ anomalous maps for the two FeMo clusters (2.18 Å resolution) [12]. It can be seen that there are significant anomalous densities at all sulfur sites in the two clusters (3.3–8.3 $\sigma$), including the $\mu_{2}$ bridging sites. However, the density is somewhat lower at the sites modelled by N_2_ (in the original publication [12], the maps were shown at a $\sigma$-level just before densities are seen at the sites modelled as N_2_). On the other hand, the very large anomalous density on the Mo ion does not reach the three coordinated sulfide ions (S1B, S3B and S4B) until a level at which the anomalous density at the putative N_2_ sites is large. In particular, the anomalous densities at the putative N_2_ sites are appreciably higher than the noise level (the first peaks at random positions appear at 3.1 $\sigma$).

It is also notable that the anomalous densities are significantly larger for the FeMo cluster in chain C than for the cluster in chain A (by 1.4 $\sigma$ units on average). This difference is connected to appreciably higher B factors for all atoms in the FeMo cluster in chain A (average 47) than in chain B (average 34).

As for the electron density, we obtain more reliable estimates by integrating the anomalous 2*mF*_o_–*DF*_c_ electron densities within a sphere with the covalent radius of S. The results are collected in Table 4. It can be seen that the integrated anomalous density at the 3A and 5A sites of chain C (8.6 and 8.8 $e$) indeed is lower than in the S2B site (11.9 $e$) and the Fe-side sulfur atoms (S1A, S2A and S4A, 9.7–13.0 $e$). However, the integrated anomalous densities of two of the Mo-side sulfide ions (S3B and S4B) are similar or even lower, 8.2–8.8 $\sigma$. If the integrated anomalous densities are compared to those of all other S atoms in the crystal structure, the densities at the 3A and 5A sites are just below the average (− 0.2 and − 0.1 $\sigma$). If they are instead compared to the other eight S atoms in the C-chain FeMo cluster, the deviation is somewhat larger (− 0.9 and − 0.8 $\sigma$), but these deviations are far from significant.

**Table 4:** Integrated anomalous electron densities for all S atoms in the two FeMo clusters in nitrogenase, using the deposited anomalous density maps (but with the putative N_2_ molecules replaced by an S atom at a position taken from the quantum-refined structures when integrating the electron densities)

Atom	Chain A			Chain C		
	*ρ*	*ρ*_all_	*ρ*_FeMo_	*ρ*	*ρ*_all_	*ρ*_FeMo_
Cys	12.0	1.11	1.70	14.0	1.90	1.76
S2B	6.0	− 1.25	− 1.68	11.9	1.08	0.73
S3A	9.0	− 0.05	0.04	8.6	− 0.22	− 0.90
S5A	8.4	− 0.30	− 0.33	8.8	− 0.14	− 0.81
S1A	8.4	− 0.30	− 0.32	9.7	0.21	− 0.36
S2A	11.2	0.80	1.25	13.0	1.50	1.26
S4A	9.4	0.09	0.24	11.5	0.92	0.52
S1B	7.3	− 0.74	− 0.95	9.7	0.22	− 0.35
S3B	8.1	− 0.43	− 0.51	8.2	− 0.36	− 1.08
S4B	10.0	0.31	0.56	8.8	− 0.12	− 0.78
Av		9.2	9.0		9.2	10.4
SD		2.5	1.8		2.5	2.0

$\rho$ is the raw integrated anomalous electron density in units of $e$. In *ρ*_all_ and *ρ*_FeMo_, this density is presented in $\sigma$ units compared to the average and standard deviation over all S atoms in the crystal structure or the S atoms within the same cluster, respectively (the corresponding average and standard deviations are given in the last two lines of the table

Likewise, it can be seen that the integrated anomalous density at the 2B site in chain A (6.0 $e$) is lower than in the 3A and 5A sites (8.4–9.0 $e$) and actually lower than for any of the sulfur atoms in the cluster (7.3–12.0 $e$). Compared to all the (140) other S atoms in the crystal structure, it is rather small (− 1.3 $\sigma$), but there are 16 atoms with lower integrated anomalous densities (11%). Compared to the other nine S atoms in the same FeMo cluster, it deviates by − 1.7 $\sigma$, corresponding to a significance of 0.87. Thus, it is not unexpected that one of ten sulfur atoms shows a deviation of that level (the Cys sulfur atom of the same cluster shows the same deviation in the opposite direction).

Thus, we conclude that not even the anomalous densities give any strong support to the suggestion that the sulfur ions are replaced by N_2_-derived ligands. In particular, there is no doubt that all sites are significantly occupied with sulfur ions (cf. Figure 6).

### Distances to the homocitrate ligand

In the original publication [12], the authors reported large differences in the Mo–O distances to the homocitrate ligand in the two FeMo clusters. In chain A, the Mo–O distance to the alcohol group on homocitrate (O7) is 2.35 Å, whereas the distance to the carboxylate oxygen (O6) is 2.73 Å. Both distances are appreciably longer than in accurate crystal structures of the resting state of the FeMo cluster, e.g. 2.18 and 2.21 Å, respectively, in the 3U7Q structure [4]. In the FeMo cluster in chain C, the distances are similar, but opposite: the distance to the alcohol group is long, 2.74 Å, whereas the distance to the carboxylate group is 2.32 Å (according to the 6UG0 structure; the article reports a distance of 2.0 Å [12]). The authors suggested that these differences may be mechanistically significant, possibly representing protonation events of the hydroxyl group [12].

Interestingly, our quantum-refinement calculations give no support to this suggestion as can be seen in Table 5. All calculations give nearly the same Mo–O distances for the hydroxyl and carboxylate groups (within 0.03 Å). Moreover, they are short in all structures, 2.05–2.16 Å. They are shortest with two N_2_ ligands (2.05–2.06 Å), intermediate with one N_2_ group (2.08–2.12 Å) and longest with only sulfide ligands (2.10–2.16 Å), reflecting the net charge of the cluster. This is confirmed by the N_2_ calculations with a net QM charge of − 3, which give 0.01–0.04 Å longer Mo–O bonds than the corresponding calculations with a net charge of − 1.

**Table 5:** Mo–O distances (Å) in the various structures

Ligands		$q$	Mo–O7	Mo–O6
2B			Chain A	
Original structure			2.35	2.73
S^2–^		− 3	2.13	2.16
N_2_		− 1	2.10	2.12
N_2_		− 3	2.13	2.16

3A	5A		Chain C	
Original structure			2.74	2.32
N_2_	N_2_	+ 1	2.06	2.05
S^2–^	N_2_	− 1	2.09	2.09
S^2–^	N_2_	− 3	2.10	2.10
S^2–^	NNH_2_	− 1	2.08	2.09
N_2_	S^2–^	− 1	2.08	2.08
N_2_	S^2–^	− 3	2.10	2.09
NNH_2_	S^2–^	− 1	2.08	2.08
S^2–^	S^2–^	− 3	2.10	2.10

O7 is the alcohol and O6 the carboxylate atom of homocitrate (cf. Fig. 1). $q$ is the net charge of the QM system

For the FeMo cluster in chain C, the electron-density difference maps in Fig. 5 show no significant features around the Mo ion and the homocitrate ligand. In particular, the structure is strongly improved compared to the original crystal structures, shown in Fig. 3. This is also reflected in the RSZD scores in Table 3. This indicates that the original crystal structure contains distances that are wrong by $\sim$ 0.6 Å. For the FeMo cluster in chain A, the situation is slightly less clear, because the quantum-refined structures show strong negative densities around the Mo ion (cf. Figure 4). However, the structure is still appreciably better than the original crystal structure in Fig. 2, as is confirmed by the RSZD scores in Table 2.

Finally, we note that the quantum-refined structures with no N_2_ ligands reproduce the metal–metal and metal–ligand distances in the high-resolution crystal structure of the resting state [4] within 0.03–0.08 Å with maximum deviations of 0.08–0.20 Å. Thus, there are no indications of any significant changes in the structure compared to the resting state.

## Conclusions

In this study, we have made a critical evaluation of the recent crystal structure of Mo nitrogenase [12], suggested to show that the $\mu_{2}$ bridging sulfide ligands are replaced by substrate N_2_-derived ligands. We make several important observations.

- The crystal structure is of poor and uneven quality, with a strong anisotropy.
- The electron-density maps (Figs. 2 and 3) do not give any support for the binding of N_2_ to the cluster and there is no indication that the electron density for the putative N_2_ ligands is significantly lower than for the other sulfide sites. On the contrary, the suggested N_2_ ligands give rise to strong and highly significant positive difference densities in both clusters (Figs. 2b and 3b).
- The suggestion that the $\mu_{2}$ bridging ligands are diatomic is probably an artefact caused by the strong anisotropy of the data.
- Quantum-refinement calculations with different interpretations of the atoms in the $\mu_{2}$ positions (Tables 2 and 3) show that sulfide ligands always give better RSZD scores than N_2_ or N_2_H_2_ ligands. This is also supported by difference electron-density maps (Figs. 4 and 5).
- Likewise, the quantum-refinement calculations give no support to the suggestion that the homocitrate ligand should bind monodentately in the crystal structure.
- Anomalous electron-density maps, obtained at 7100 eV (Fig. 6), show that the anomalous density indeed is somewhat lower at the putative N_2_ sites, but it is still significant and actually larger than for the sulfides on the Mo-side of the cluster. Moreover, a statistical analysis of the anomalous densities (Table 4), show that the densities are not lower than what could be expected by random variations.

Consequently, we conclude that there is no convincing evidence that the crystal structure should show any bound N_2_-derived ligands. Instead, a standard resting state with nine sulfide ligands seems to be a better interpretation.

## Supplementary Information

Below is the link to the electronic supplementary material.

Supplementary file1 (PDF 9232 KB)

## Note added in proof

After acceptance of this article, a comment to ref. 12 has been published (J. W. Peters et al., Science 10.1126/science.abe5481 (2021)) with partly similar arguments as ours, as well as a response from the original authors W. Kang et al., Science 10.1126/science.abe5856 (2021).
