# Supplementary material for: Critical evaluation of a crystal structure of nitrogenase with bound N2 ligands
Source: J Biol Inorg Chem. 2021 Mar 13;26(2):341–53. doi: 10.1007/s00775-021-01858-8 (PMC8068654; doi:10.1007/s00775-021-01858-8)
Supplement: Supplementary file 1 — Supplementary file1 (PDF 9232 KB) [file 775_2021_1858_MOESM1_ESM.pdf]

Supplementary material of: Critical evaluation of a crystal structure of nitrogenase with bound N_2 ligands

Justin Bergmann[a], Esko Oksanen[b], Ulf Ryde*[a]

[a]*Department of Theoretical Chemistry, Lund University, Chemical Centre, P.O. Box 124, SE-221 00 Lund, Sweden. E-mail: Ulf.Ryde@teokem.lu.se*
[b]*European Spallation Source ESS ERIC*

Preprint submitted to J. Biol. Inorg. Chem. *January, 2021*

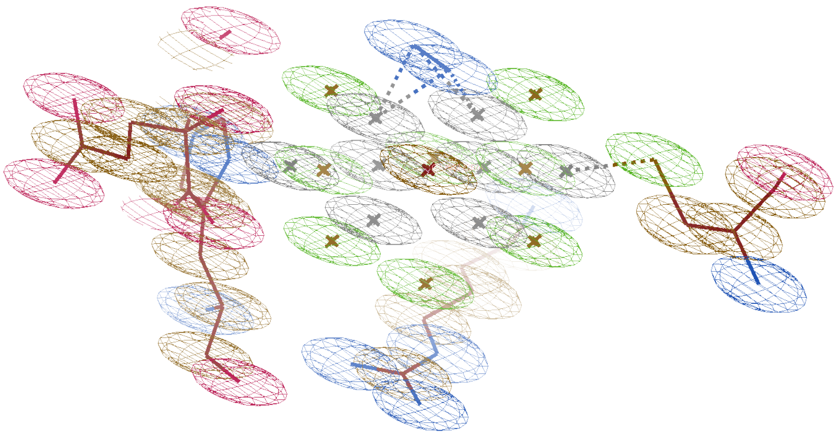

Figure S1: Anisotropic B-factors for the FeMo-cluster in chain A at 50% probability

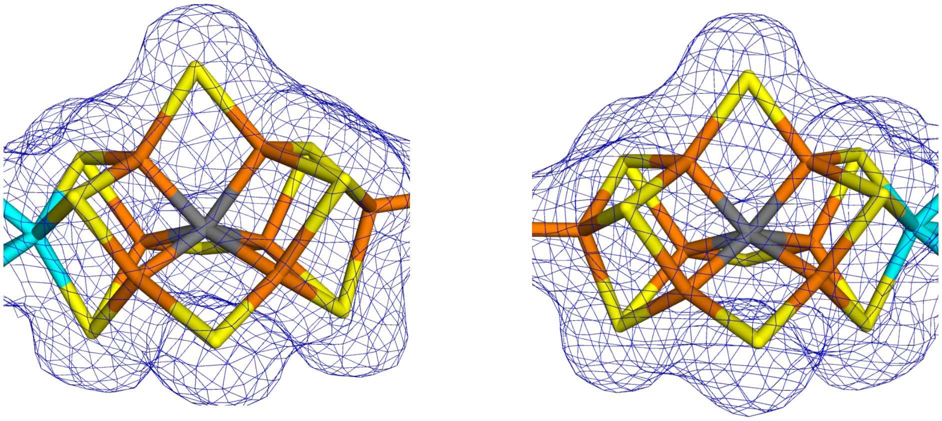

Figure S2: Electron-density maps around the active-side FeMo cluster in chain A of nitrogenase modelled with a sulfide ion in site 2B. The figures show two orientations of the cluster. The $2mF_\mathrm{o} - DF_\mathrm{c}$ map is contoured at 1 σ (blue).

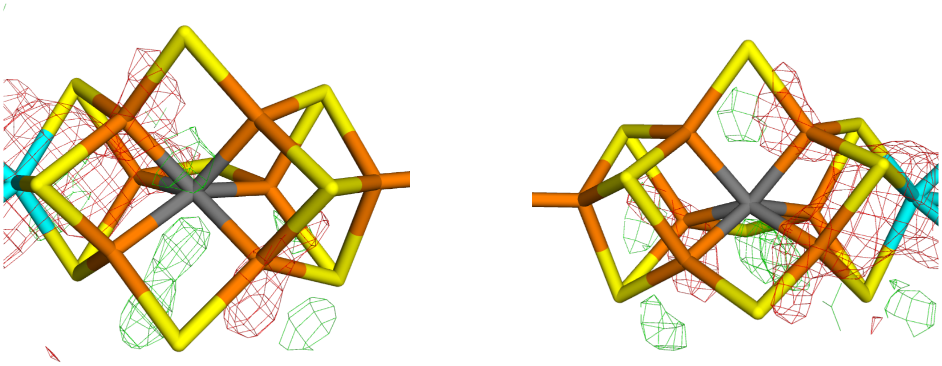

Figure S3: Electron-density difference maps around the active-side FeMo cluster in chain A of nitrogenase modelled with sulfide in site 2B. The figures show two orientations of the cluster. The $mF_o - DF_c$ difference map is contoured at 3 σ (green) and –3 σ (red).

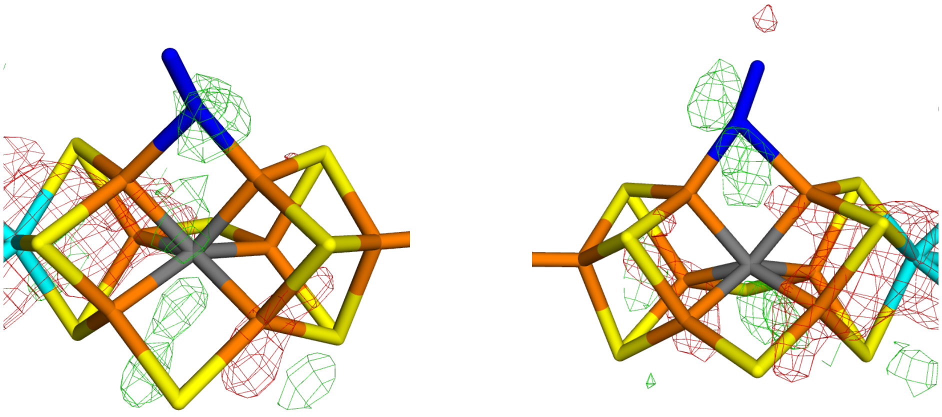

Figure S4: Electron-density difference maps around the active-side FeMo cluster in chain A of nitrogenase modelled with N_2 in site 2B. The figures show two orientations of the cluster. The $mF_o - DF_c$ difference map is contoured at 3 σ (green) and –3 σ (red).

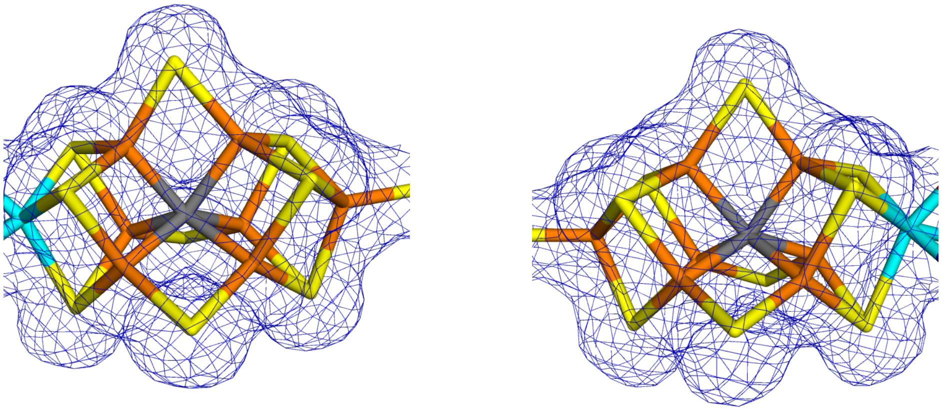

Figure S5: Electron-density maps around the active-side FeMo cluster in chain C of nitrogenase modelled with sulfide ions in both sites 3A and 5A. The figures show two orientations of the cluster. The $2mF_\mathrm{o} - DF_\mathrm{c}$ map is contoured at 1 σ (blue).

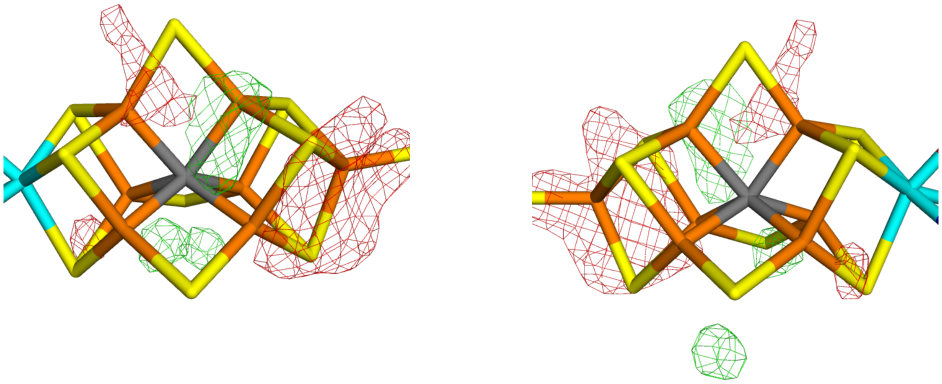

Figure S6: Electron-density difference maps around the active-side FeMo cluster in chain C of nitrogenase modelled with sulfide ions in both sites 3A and 5A. The figures show two orientations of the cluster. The $mF_\mathrm{o} - DF_\mathrm{c}$ difference map is contoured at 3 σ (green) and –3 σ (red).

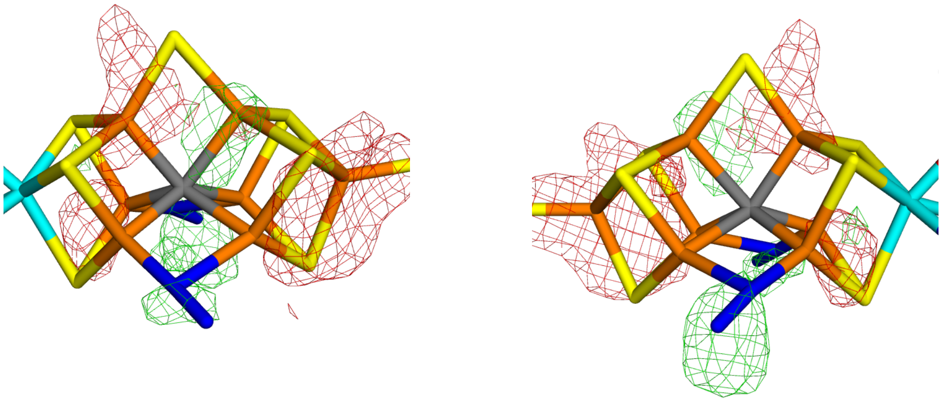

Figure S7: Electron-density difference maps around the active-side FeMo cluster in chain C of nitrogenase modelled with N_2 in both sites 3A and 5A. The figures show two orientations of the cluster. The $mF_o - DF_c$ difference map is contoured at 3 σ (green) and –3 σ (red).

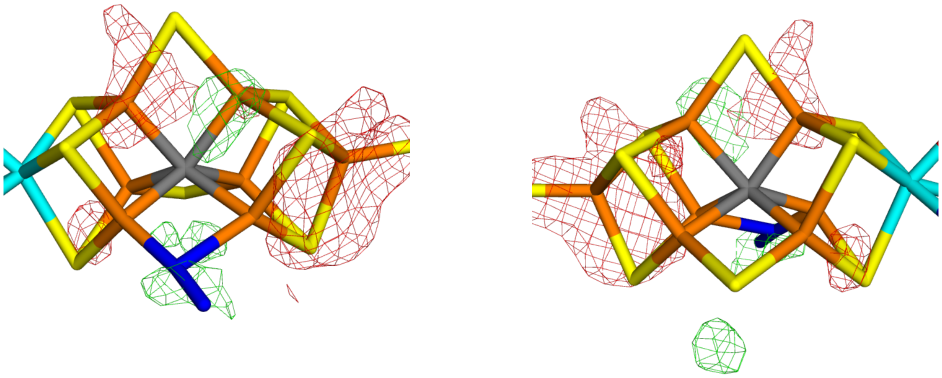

Figure S8: Electron-density difference maps around the active-side FeMo cluster in chain C of nitrogenase modelled with N_2 in site 3A and sulfide in site 5A. The figures show two orientations of the cluster. The $mF_o - DF_c$ difference map is contoured at 3 σ (green) and –3 σ (red).

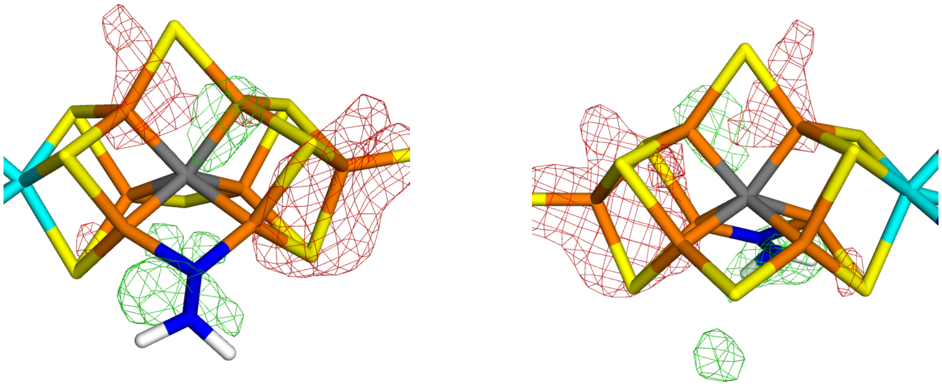

Figure S9: Electron-density difference maps around the active-side FeMo cluster in chain C of nitrogenase modelled with N_2H_2 in site 3A and sulfide in site 5A and sulfide in site 3A. The figures show two orientations of the cluster. The $mF_o - DF_c$ difference map is contoured at 3 σ (green) and –3 σ (red).

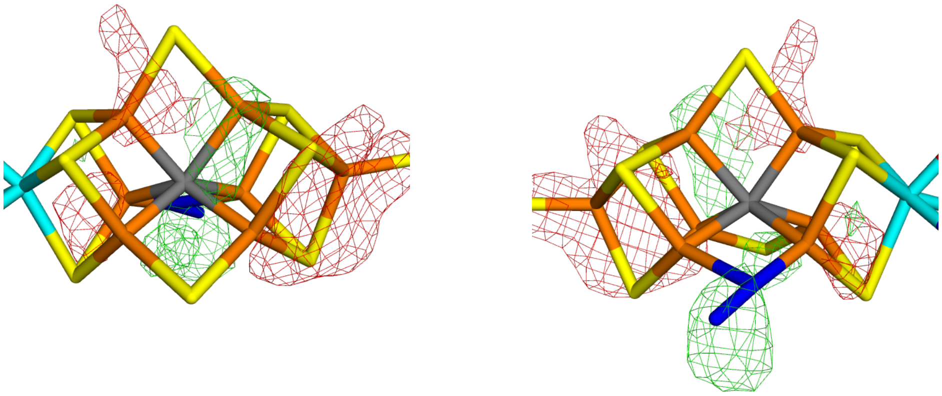

Figure S10: Electron-density difference maps around the active-side FeMo cluster in chain C of nitrogenase modelled with N_2 in site 5A and sulfide in site 3A. The figures show two orientations of the cluster. The $mF_o - DF_c$ difference map is contoured at 3 σ (green) and –3 σ (red).

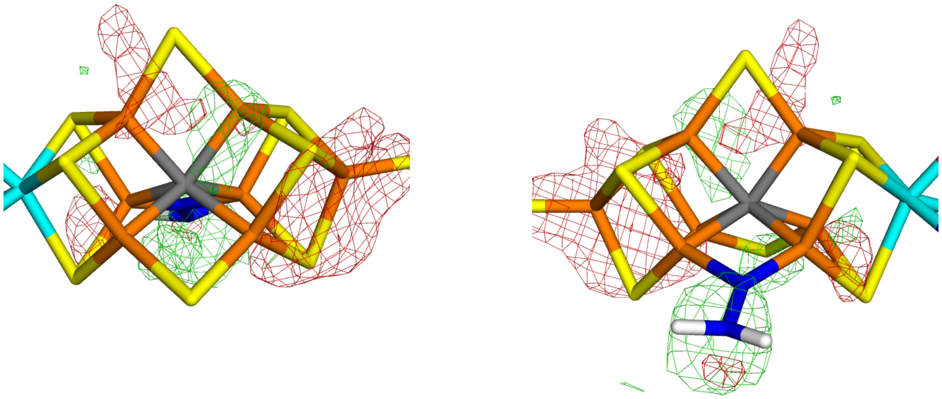

Figure S11: Electron-density difference maps around the active-side FeMo cluster in chain C of nitrogenase modelled with N_2H_2 in site 5A and sulfide in site 3A. The figures show two orientations of the cluster. The $mF_\mathrm{o} - DF_\mathrm{c}$ difference map is contoured at 3 σ (green) and −3 σ (red).

Coordinates of the QM systems in xyz format

```
81
Chain C with N2 in both 3A and 5A
C 29.317 125.828 122.188
H 29.18 124.764 121.922
H 28.334 126.328 122.145
N 30.217 126.429 121.21
H 30.908 125.791 120.786
C 29.908 127.514 120.488
N 29.097 128.447 121.033
H 28.672 129.178 120.465
H 28.939 128.468 122.039
N 30.385 127.656 119.235
H 30.451 128.566 118.779
H 30.826 126.864 118.758
H 35.86 130.084 112.803
C 35.047 129.759 113.485
H 34.167 130.417 113.383
H 35.41 129.751 114.527
S 34.576 128.013 112.938
H 39.16 124.61 117.912
C 38.625 124.379 118.849
H 39.329 123.935 119.574
H 37.803 123.67 118.632
N 38.105 125.644 119.39
H 37.864 126.378 118.718
C 37.608 125.752 120.632
N 37.527 124.646 121.408
H 37.181 123.783 120.962
H 37.237 124.759 122.381
N 37.252 126.957 121.123
H 37.47 127.816 120.617
H 36.607 127.028 121.91
H 34.836 120.74 122.327
C 34.813 120.035 121.483
C 35.275 118.731 121.457
H 35.833 118.136 122.179
N 34.095 120.277 120.322
C 34.072 119.141 119.623
H 33.565 118.988 118.67
N 34.808 118.199 120.261
H 34.921 117.231 119.958
O 31.983 116.764 122.176
C 32.046 117.867 122.812
O 32.852 117.63 123.809
C 31.595 119.291 122.778
H 31.501 119.62 123.826
H 32.451 119.835 122.334
C 30.329 119.546 121.952
H 30.183 118.751 121.197
H 29.443 119.546 122.611
C 30.422 120.884 121.204
O 31.222 120.708 120.028
H 30.032 120.392 119.016
C 31.152 121.877 122.14
O 32.356 122.21 121.69
O 30.67 122.242 123.201
C 29.01 121.429 120.849
H 28.399 121.501 121.762
H 29.124 122.447 120.423
C 28.282 120.581 119.795
O 29.068 120.21 118.767
O 27.101 120.32 119.834
C 33.488 124.488 117.281
S 33.872 124.69 114.068
S 32.825 121.366 117.475
S 32.203 127.229 115.859
S 30.654 123.641 115.748
S 31.191 123.867 119.413
S 35.652 126.814 116.248
S 34.836 123.24 119.927
FE 34.062 126.9 114.754
FE 32.366 124.973 115.569
FE 33.795 126.498 117.305
FE 35.004 124.632 115.909
FE 34.472 122.854 117.817
FE 31.869 123.344 117.403
FE 33.219 124.57 119.19
MO 32.764 122.035 119.687
N 35.785 122.754 116.618
N 36.749 122.742 115.997
N 33.445 126.316 119.441
N 33.488 127.44 119.698
```

79
Chain C with S in both 3A and 5A
H 29.729 125.843 123.216
C 29.401 125.761 122.156
H 29.409 124.679 121.92
H 28.36 126.128 122.061
N 30.275 126.413 121.197
H 30.972 125.777 120.733
C 29.954 127.499 120.485
N 29.065 128.411 121.03
H 28.502 128.931 120.352
H 28.524 128.075 121.827
N 30.506 127.713 119.285
H 30.469 128.656 118.894
H 31.449 127.217 119.093
H 35.867 130.084 112.801
C 35.061 129.752 113.486
H 34.208 130.459 113.439
H 35.451 129.709 114.52
S 34.576 128.032 112.896
H 39.133 124.605 117.886
C 38.531 124.361 118.78
H 39.15 123.861 119.546
H 37.691 123.708 118.45
N 38.046 125.64 119.317
H 37.561 126.222 118.611
C 37.549 125.741 120.575
N 37.467 124.632 121.378
H 36.904 123.855 120.946
H 37.105 124.851 122.31
N 37.123 126.97 121.065
H 37.3 127.725 120.393
H 36.103 126.963 121.269
H 34.836 120.74 122.332
C 34.811 120.035 121.49
C 35.232 118.719 121.478
H 35.68 118.091 122.246
N 34.113 120.297 120.328
C 34.055 119.165 119.642
H 33.538 119.042 118.689
N 34.77 118.195 120.273
H 34.649 117.197 120.1
O 31.957 116.723 122.084
C 32.164 117.85 122.95
O 32.943 117.675 123.93
C 31.661 119.285 122.856
H 31.545 119.664 123.884
H 32.497 119.848 122.402
C 30.403 119.501 122.005
H 30.328 118.678 121.267
H 29.499 119.462 122.647
C 30.436 120.841 121.242
O 31.196 120.679 120.042
H 30.071 120.396 119.099
C 31.143 121.87 122.163
O 32.331 122.217 121.746
O 30.609 122.248 123.215
C 29.009 121.377 120.897
H 28.393 121.433 121.809
H 29.138 122.402 120.489
C 28.291 120.566 119.819
O 29.081 120.203 118.808
O 27.091 120.317 119.832
C 33.493 124.529 117.249
S 33.867 124.695 114.058
S 32.825 121.349 117.481
S 32.199 127.24 115.852
S 30.635 123.646 115.735
S 31.163 123.851 119.441
S 35.684 126.855 116.241
S 34.847 123.202 119.941
FE 34.059 126.901 114.759
FE 32.376 124.974 115.577
FE 33.804 126.524 117.322
FE 35.013 124.644 115.923
FE 34.564 122.845 117.761
FE 31.881 123.348 117.403
FE 33.227 124.636 119.226
MO 32.763 122.046 119.694
S 36.194 122.786 116.311
S 33.454 126.882 119.533

80
Chain A with N2 in 2B
H 32.126 125.937 180.318
C 32.392 125.782 181.381
H 33.454 126.047 181.543
H 32.269 124.702 181.581
N 31.54 126.503 182.331
H 30.779 125.948 182.773
C 31.904 127.596 183.01
N 32.812 128.466 182.475
H 33.272 128.208 181.603
H 33.358 129.046 183.111
N 31.384 127.847 184.223
H 30.519 127.309 184.494
H 31.402 128.805 184.575
H 25.93 130.105 190.571
C 26.764 129.791 189.89
H 26.372 129.682 188.862
H 27.567 130.553 189.905
S 27.398 128.173 190.531
H 22.73 124.61 185.47
C 23.416 124.345 184.649
H 24.205 123.675 185.05
H 22.86 123.862 183.827
N 24.008 125.595 184.147
H 24.386 126.234 184.861
C 24.416 125.78 182.886
N 24.444 124.741 182.02
H 24.704 124.961 181.057
H 24.908 123.887 182.392
N 24.715 127.032 182.439
H 25.561 127.101 181.862
H 24.663 127.78 183.137
H 26.948 120.834 181.289
C 26.937 120.149 182.151
C 26.48 118.849 182.173
H 25.935 118.263 181.434
N 27.709 120.377 183.291
C 27.765 119.221 183.941
H 28.313 119.05 184.867
N 27.021 118.276 183.311
H 26.966 117.294 183.575
O 28.782 117.517 179.535
C 29.511 118.02 180.455
O 29.925 117.136 181.311
C 29.972 119.401 180.872
H 29.225 119.726 181.615
H 29.876 120.106 180.022
C 31.34 119.561 181.524
H 32.144 119.574 180.764
H 31.536 118.743 182.239
C 31.326 120.907 182.3
O 30.543 120.751 183.487
H 31.684 120.386 184.428
C 30.651 121.944 181.382
O 31.199 122.315 180.349
O 29.456 122.329 181.779
C 32.757 121.409 182.641
H 32.669 122.437 183.048
H 33.37 121.449 181.726
C 33.463 120.567 183.708
O 34.653 120.31 183.684
O 32.656 120.168 184.701
C 28.266 124.57 186.203
S 27.907 124.671 189.466
S 28.968 121.274 186.053
S 29.711 127.182 187.696
S 25.808 122.829 187.267
S 30.712 123.792 184.067
S 26.168 126.962 187.229
S 26.964 123.343 183.545
S 28.418 126.773 184.032
N 30.856 123.98 187.741
N 31.902 123.532 187.887
FE 27.785 126.808 188.785
FE 29.367 125.014 187.853
FE 28.057 126.509 186.171
FE 26.755 124.749 187.539
FE 27.412 122.86 185.757
FE 29.789 123.303 186.069
FE 28.668 124.562 184.267
MO 29.007 122.143 183.846

79
Chain A with S in 2B
H 32.116 125.928 180.321
C 32.355 125.739 181.388
H 32.193 124.656 181.555
H 33.425 125.963 181.574
N 31.503 126.449 182.342
H 30.769 125.857 182.806
C 31.866 127.53 183.041
N 32.787 128.417 182.508
H 33.306 128.069 181.699
H 33.383 128.884 183.195
N 31.338 127.77 184.244
H 30.424 127.257 184.483
H 31.388 128.716 184.626
H 25.929 130.108 190.573
C 26.761 129.794 189.893
H 26.36 129.655 188.871
H 27.543 130.583 189.877
S 27.396 128.197 190.574
H 22.727 124.618 185.481
C 23.442 124.36 184.68
H 24.227 123.7 185.114
H 22.923 123.867 183.84
N 24.029 125.618 184.201
H 24.511 126.173 184.933
C 24.461 125.799 182.938
N 24.483 124.762 182.052
H 24.812 125.039 181.122
H 25.036 123.943 182.419
N 24.856 127.055 182.516
H 25.848 127.055 182.209
H 24.767 127.752 183.265
H 26.951 120.833 181.284
C 26.942 120.145 182.143
C 26.527 118.832 182.145
H 26.066 118.226 181.365
N 27.694 120.387 183.292
C 27.787 119.228 183.926
H 28.34 119.075 184.852
N 27.072 118.262 183.286
H 27.158 117.263 183.46
O 28.758 117.522 179.497
C 29.495 117.933 180.457
O 29.921 117.083 181.334
C 29.954 119.34 180.88
H 29.225 119.654 181.643
H 29.826 120.055 180.039
C 31.33 119.537 181.504
H 32.123 119.559 180.729
H 31.539 118.713 182.21
C 31.326 120.886 182.279
O 30.559 120.737 183.479
H 31.665 120.402 184.391
C 30.659 121.93 181.362
O 31.235 122.306 180.335
O 29.474 122.32 181.742
C 32.759 121.393 182.62
H 32.656 122.422 183.023
H 33.377 121.431 181.708
C 33.46 120.563 183.697
O 34.66 120.305 183.679
O 32.654 120.173 184.683
C 28.291 124.57 186.217
S 27.907 124.662 189.492
S 28.961 121.271 186.045
S 29.691 127.246 187.685
S 31.14 123.77 187.739
S 25.788 122.818 187.264
S 30.712 123.792 184.042
S 26.146 126.97 187.224
S 26.967 123.348 183.543
S 28.416 126.791 184.018
FE 27.786 126.806 188.789
FE 29.399 125.032 187.867
FE 28.055 126.509 186.17
FE 26.765 124.749 187.535
FE 27.419 122.872 185.761
FE 29.823 123.3 186.08
FE 28.668 124.569 184.269
MO 29.008 122.148 183.847
